# Taguchi optimization and modelling of stir casting process parameters on the percentage elongation of aluminium, pumice and carbonated coal composite

**DOI:** 10.1038/s41598-023-29839-8

**Published:** 2023-02-20

**Authors:** Tanimu Kogi Ibrahim, Danjuma Saleh Yawas, Bashar Dan-asabe, Adetayo Abdulmumin Adebisi

**Affiliations:** 1 Faculty of Engineering, Federal University Wukari, Taraba, Nigeria; 2 Mechanical Engineering Department, Faculty of Engineering, Ahmadu Bello University, Zaria, Nigeria; 3 Shell JV Professorial Chair Office, Mechanical Engineering Department, Ahmadu Bello University, Zaria, Nigeria; 4 Metallurgical and Materials Engineering Department, Faculty of Air Engineering, Air Force Institute of Technology, Kaduna, Nigeria; 5 Metallurgical and Materials Engineering Department, Faculty of Engineering, Ahmadu Bello University, Zaria, Nigeria

**Keywords:** Materials science, Engineering, Mechanical engineering

## Abstract

Aluminium matrix composites, which are a subclass of metal matrix composites, have characteristics including low density, high stiffness and strength, better wear resistance, controlled thermal expansion, greater fatigue resistance, and improved stability at high temperatures. The scientific and industrial communities are interested in these composites because they may be used to manufacture a broad variety of components for cutting-edge applications. This has study observed how the stirring speed, processing temperature, and stirring duration of the stir casting process affected the percentage elongation of Al-Pumice (PP)-Carbonized Coal Particles (CCP) hybrid composites. It also looked at the optimal weight of these natural ceramic reinforcements using the Taguchi optimization technique. While optimizing the percentage elongation property, the hard compound such as silica, iron oxide, and alumina, were discovered during the characterisation of the reinforcement, showing that PP and CCP can be used as reinforcement in metal matrix composite. The percentage of elongation of the hybrid composite was shown to be most affected by the PP, followed by processing temperature, stirring speed, CCP, and stirring time, using stir casting process parameter optimization. It was observed at 2.5 wt% of pumice particles, 2.5 wt% of carbonated coal particles, 700 °C processing temperature, 200 rpm stirring speed, and 5 min stirring time, the optimum percentage of elongation was discovered to be 5.6%, which is 25.43% lower than the percentage elongation of Al-alloy without reinforcing. The regression study developed a predictive mathematical model for the percentage elongation (PE) as a function of the stir casting process parameters and offered a high degree of prediction, with R-Square, R-Square (adj), and R-Square (pred) values of 91.60%, 87.41%, and 79.32% respectively.

## Introduction

Currently, stronger, lighter, and more affordable materials are needed for cutting edge applications^1^. To achieve these criteria, researchers are now concentrating on developing hybrid composites with a strong strength-to-weight ratio^2^. Aluminium alloy is the most widely used alloy to develop the hybrid composite due to its high strength-to-weight ratio, thermal conductivity, workability, casting, and forging properties. But aluminium alloys have certain disadvantages, such as low stiffness, toughness, fatigue resistance, a high coefficient of thermal expansion, and inadequate tribological characteristics. Among the most effective ways of achieving improvements in the properties of aluminium alloys is the creation of hybrid composites with two or more types of reinforcement. Hybrid composites provide several benefits over monolithic, alloy, and composite materials, including a high strength-to-weight ratio, superior corrosion and wear resistance, strength and stiffness, low thermal conductivity and thermal expansion, low weight, and improved impact and flexural characteristics. lower composite cost overall^3,4^. Hybrid materials are made up of one matrix and two or more reinforcing elements^5^. They are manufactured using a variety of techniques, including powder metallurgy, stir casting, two-step stir casting, and squeeze casting^6^, to achieve the desired mechanical properties and tribological behaviour: high specific strength, including stiffness, density, microhardness, low coefficient of thermal expansion, high thermal resistance, and good damping capacity^7^.

Ceramic particles like pumice and carbonized coal particles have proved to significantly improve the mechanical characteristics of aluminium and its alloys when used as reinforcement^8^. Aluminium’s hardness, yield strength, and tensile strength are increased, but ductility and percentage elongation are reduced by the addition of particles such as alumina, SiC, B_4_C, etc.^9^. When compared to the fundamental materials used in ceramics, pumice exhibits some chemically comparable qualities^10^. The remaining 60 to 75% of the material, which is mostly composed of Al_2_O_3_ and SiO_2_, is composed of these two oxides^8^. When its composition is combined with the sizes of known deposits, which total billions of tons, pumice, which can be in its particle form (i.e., pumice particle-PP), has the potential to be used as a ceramic raw material^10^. Because of their numerous beneficial attributes, such as their pozzolanic properties, tiny particle size, abrasive nature, and mineralogy, carbonated coal particles (CCP) also have the potential for significant usage in the field of ceramics^11,12^. To extend service life while reducing weight, great effort has been put into improving the mechanical characteristics of composites comprised of an aluminium matrix^13^. Even if the performance of other mechanical qualities has improved, the fundamental drawback of ceramic reinforcing materials is the decrease in the percentage elongation of the AMCs^14^. Aluminium composites' hardness and brittleness may increase if ceramic particles are added to the alloy^5^. Utilizing such composites has become challenging due to this property. Investigations of the aluminium alloy's reinforcements are necessary to assess its performance in certain applications and to get beyond these restrictions.

Stir-casting techniques are currently the simplest and most commercially viable method of manufacturing metal matrix composites. This method entails mechanically mixing of the reinforcement particulate in a molten metal bath and then transferring the mixture to a formed mould until it solidifies fully^15^. The experimental set-up includes a resistance-heating furnace to melt the base metal, a feeding mechanism for ceramic particles, and a mechanical stirrer coupled to an electrical motor to mix the pre-heated particles with the matrix liquid^16^. The most recent intriguing breakthrough is the double stir casting, or two-step mixing process. In this process, the matrix material is heated above its liquidus temperature, then melt is cooled to a temperature between the liquidus and solidus points (semi-solid state). Preheated reinforcement particles are added and mixed at this stage. The slurry is then heated to a complete liquid state and thoroughly mixed once more. The key problems with the stir casting method are reinforcement particle agglomeration, porosity/gas entrapment, reaction viscosity and segregation caused by particle settling during solidification and particle agglomeration. For uniform dispersion of reinforcement particles, choosing the settings for the stirring process presents significant hurdles^17^.

The impact of process variables on the dispersion of reinforcement particles has been extensively researched. The crucial variables that were tracked were the stirring duration, processing temperature, and pace. According to Singh et al. a 550 rpm stirring speed, a 45° blade angle stirrer, and a 6-min stirring period are the ideal settings for uniformly dispersing particles in casted samples^18^. Moses et al.^19^ found that the tensile strength of the cast composite was at its peak while stirring at 300 rpm for 15 min, at 30° blade angle. According to Prabu et al.^20^ more uniform dispersion of particles was seen in the cast specimens at a stirring speed of 600 rpm and a stirring period of 10 min. In the same specimen, they noted more hardness as well. Before pouring the composite into the cavity of the mould, homogeneous dispersion of the particles is desired. The phenomena of flow and solidification would cause inhomogeneity in the poured melt to increase. Gravity and the changing kinetic circumstances that arise during the transition from a liquid to a solid impact the ultimate dispersion of particles during solidification^21^.

Studies have improved the input parameters that result in an output response based on the Taguchi approach of optimization^22^. Using the loss function of this approach, performance measures that deviate from the desired target value are calculated. The value of this loss function is used to calculate the signal-to-noise (S/N) ratio. In general, three categories are used to categorize performance: "smaller the better," "nominal the better," and "higher the better" statistics^23^. The goal and novelty of this study are to, for the first time, apply the Taguchi optimization approach to optimize the weight composition of natural ceramic reinforcement (pumice and carbonated coal particles), as well as the impact of stir casting process parameters (stirring speed, processing temperature, and stirring time) on a "higher is better" optimal percentage elongation of Al-PP-CCP hybrid composites.

## Materials and methods

### Materials

Aluminium powder, pumice, and coal were employed in the production of the Al-PP-CCP hybrid composites. While coal was sourced from the Dangote coal mine in Effeche-Akpalli, Benue State, Nigeria, pumice was mined locally underground at mining locations in Biu, Borno State, Nigeria.

### Aluminium (AA6061) alloy

Aluminium 6061 is part of the 6xxx series of aluminium alloys, which have magnesium and silicon as the primary alloying elements. It has a good strength-to-weight ratio, thermal conductivity, weldability, workability, casting, machinability, forging properties, and corrosion resistance. Its applications range from automobile and aerospace components to food and beverage packaging, electronic products, etc.

### Production of pumice particle

**Figure 1:**
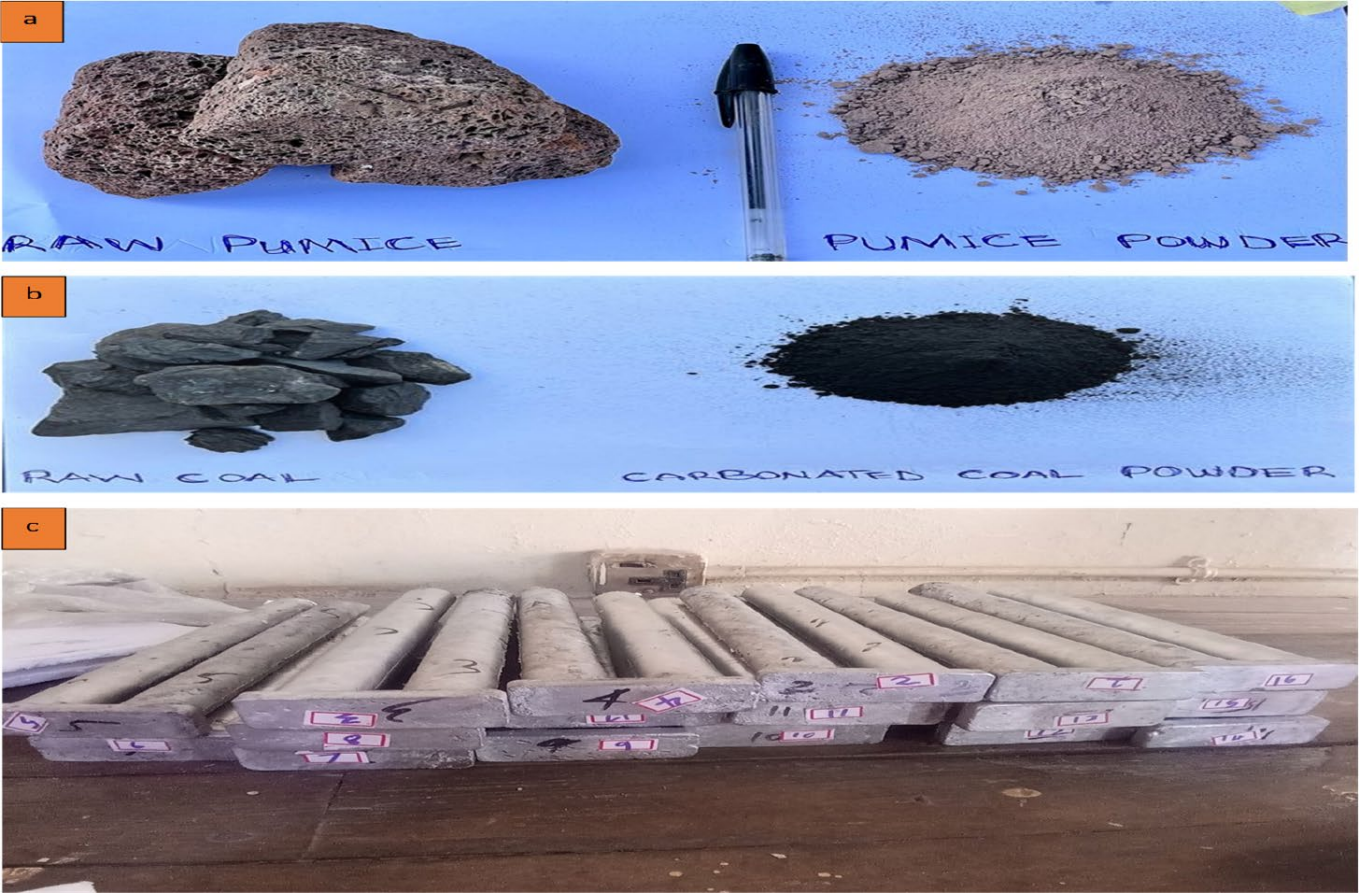
Reinforcements and cast composite (**a**) raw and powder pumice (**b**) raw and powder carbonated coal (**c**) cast hybrid aluminium composite.

To remove any moisture and grime, the pumice was washed and dried in an oven at 100 °C for 48 h after it was extracted from underground local mines in Borno State, Nigeria. The aggregated lumps were then processed into fine powders by first grinding with a lab mortar and pestle. This technique of manufacture is consistent with research by^24,25^. Pumice particles (PP) of a size of 90 μm were obtained by further sieving the pumice powder. Figure 1a shows the produced carbonated coal particles.

### Production of carbonized coal particles

Using a crushing machine, the coal that was recovered from the Dangote coal mine was first broken into smaller lumps. The lumps were placed in a graphite crucible and heated to 1100 °C for 8 h in an electric furnace without any air. The carbonized coal was cleaned with water to eliminate impurities and dried to minimize moisture content after being normalized in the oven. The carbonized coal was then pulverized and crushed in a laboratory mortar and pestle to turn the large lumps of coal into tiny granules. The studies by^26,27^ support this technique. To achieve carbonized coal particles (CCP) of 90 μm size, the produced carbonized coal underwent further sieving as. Figure 1b shows the produced carbonated coal particles.

### Production of the Al-PP-CCP hybrid composites

Al-PP-CCP hybrid composite was created utilizing the bottom pouring stir casting method and liquid metallurgy. According to^28^, the produced PP and CCP were heated in the furnace for two hours at 500 °C with the intention of oxidizing and calcining the particle surfaces. Then, to guarantee that the alloy melts fully, the Al ingots were loaded into a crucible in an electrical furnace and heated to 690 °C (30 °C over the liquidus temperature). A heated coated skimmer was used to remove the dross that had formed on the surface of the melted aluminium. To increase the wettability between the matrix and reinforcement phases and to eliminate gases from the melt, 0.01% NaCl-KCl powder and 1 wt% magnesium (which acts as a surfactant) were added before the integration of the warmed particles according to the methods of^29,30^.

A coated stainless-steel stirrer was dropped into the furnace to agitate the melt and form a vortex while the liquid alloy cooled in the furnace to a semi-solid condition at a temperature of roughly 600 °C. At this point, within a minute, the heated particles, each with a weight composition of 2.5–10 wt%, were slowly added to the melted slurry^31,32^. After that, the composite slurry was heated to various stir casting process parameters, including stirring speed (SS) (200–500 rpm), processing temperature (PT) (700–850 °C), and stirring time (ST) (5–20 min) according to the experimental run. Additionally, according to the method of Aynalem^33^, the mould was heated to a temperature of around 550 °C before slurry was poured into the mould. Al alloy without reinforcement was also made as the control sample to compare the effects of the reinforcement. The produced hybrid aluminium composite is shown in Fig. 1c.

### Experimental design

The trials in this study were designed using Taguchi's orthogonal array (OA), following the recommendation of^34^. For this investigation, five processing factors with a four design levels were used. Table 1 displays the variables and levels employed in the production of Al-PP-CCP hybrid composites, whereas Table 2 displays the orthogonal array of L16 experimental runs produced by the Minitab statistical program.

**Table 1:** Factor and Levels of the Stir Casting Process Parameters.

Factor	Levels			
PP	2.5	5	7.5	10
CCP	2.5	5	7.5	10
SS (rpm)	200	300	400	500
PT (^o^C)	700	750	800	850
ST (min)	5	10	15	20

**Table 2:** L16 Orthogonal Array of experimental runs.

Experimental run	Factors				
	PP (wt%)	CC (wt%)	SS (rpm)	PT (^o^C)	ST (min)
1	2.5	2.5	200	700	5
2	2.5	5	300	750	10
3	2.5	7.5	400	800	15
4	2.5	10	500	850	20
5	5	2.5	300	800	20
6	5	5	200	850	15
7	5	7.5	500	700	10
8	5	10	400	750	5
9	7.5	2.5	400	850	10
10	7.5	5	500	800	5
11	7.5	7.5	200	750	20
12	7.5	10	300	700	15
13	10	2.5	500	750	15
14	10	5	400	700	20
15	10	7.5	300	850	5
16	10	10	200	800	10

### Characterization

#### Scanning electron microscopy with energy dispersive X-ray analysis

The morphologies of the Al alloy, PP, and CCP powders were investigated on an ultra-high vacuum, high-resolution scanning electron microscope (SEM) with energy dispersive X-ray (EDX) operating at 16.0 kV. The samples were arranged by using a low deposition rate to sputter gold onto their surfaces.

#### X-ray fluorescence

Using a Philips X-ray fluorescence spectrometer, model PW 2400, the elemental compositions of the Al alloy, PP, and CCP were investigated. X-ray fluorescence (XRF) spectrometers were able to identify the emissive fluorescence of various substances inside the samples by differentiating between input x-rays and output gamma rays.

#### X-ray powder diffraction

The structural patterns of PP and CCP powders were determined by utilizing an X-ray powder diffraction (XRD) method using a copper tube (1.5418 A) produced at a voltage of 40 kV and a current of 30 mA in a Diffractometer Rigaku Miniflex across a 2theta range of 5°–80°^35^.

### Percentage elongation

The percentage elongation of the Al-PP-CCP hybrid composite was performed in conformity with the ASTM E8M-91 standard. Using a computerized testing machine (Zwick/Roell Z100) on specimens with a standard dimension of diameter of 12.7 mm and a gauge length of 50.8 mm, the percentage elongation was obtained. For each specimen, the elongation test was performed three times to ensure the repeatability and reliability of the data obtained^36^.

### Statistical analysis and optimization

The developed Al-PP-CCP hybrid composites' experimental percentage elongation property was analysed using Taguchi optimization, analysis of variance (ANOVA), and interaction analysis with the help of Minitab (Version 16.1, Minitab Inc.), and Origin (Version 2020, OriginLab) software. A common technique for assessing the strength of the connection between sequences is based on the signal-to-noise ratio.

In this investigation, high percentage elongation values were chosen for the overall properties of the composite. As a result, in the experimental stage, grades were created using the Taguchi optimization "larger-is-better" normalization criteria^37^. The best level of this process parameter is the one with the largest S–N ratio^22^. Equation (1) outlines the larger-is-better criteria linear data pre-processing approach that was used in this work to calculate the percentage elongation of the composite under investigation based on the S/N ratio of the function.

1 $$\eta =-10{\mathrm{log}}_{10}\left(\frac{1}{n}{\sum }_{i=1}^{n}{y}_{i}^{2}\right)$$

where n is the sample size and y_i_ is the percentage elongation of the run.

## Results and discussion

### Scanning electron microscopy with energy dispersive X-ray analysis of constituting materials

**Figure 2:**
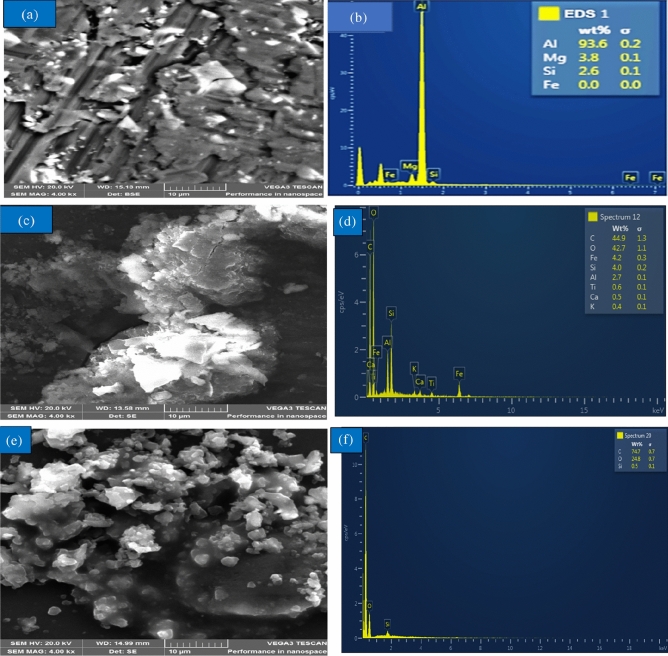
SEM and EDX of Matrix and Reinforcements: (**a**) SEM of Al Alloy, (**b**) EDX of Al Alloy, (**c**) SEM of PP, (**d**) EDX of PP, (**e**) SEM of CCP, (**f**) EDX of CCP.

The microstructure and EDS of the Al alloy, PP, and CCP are depicted in Fig. 2a,b. Primary grains of Al solid solution are depicted in Fig. 2a, along with interdimeric Al-Si eutectic regions that contain a variety of intermetallic phases, including precipitates of the intermetallic compound Mg_2_Si. This solid solution is created as a result of supercooling during solidification. The EDS analysis is depicted in Fig. 2b as having peaks for aluminium (Al), oxygen (O), carbon (C), iron (Fe), silicon (Si), calcium (Ca), sodium (Na), and magnesium (Mg). These components supported the XRF findings and verified that the alloy utilized was Al 6061 alloy.

The microstructure of the PP was disclosed in Fig. 2c, and it comprises lamellae with an amorphous structure, equally dispersed phases, and boundaries that show the material is extrusive and has a uniform distribution of pores. This indicates that one of PP's key attributes is its ability to promote stress transmission in hybrid composite applications by offering strong particulate-matrix interface adhesion with aluminium. This enhances several mechanical qualities^38^. Peaks of carbon, oxygen, iron, silicon, aluminium, titanium, calcium, and potassium may be seen in Fig. 2d. These substances are pumice-derived components, and their presence indicates the existence of SiO_2_, Al_2_O_3_, K_2_O, Fe_2_O_3_, and MgO. The XRF and XRD analyses concur with this conclusion.

The microstructure of the CCP, which is composed of angular, irregular porous spaces and a rough texture, was also disclosed in Fig. 2e. Some of the CCP's particles are spherical, while others resemble popcorn. Due to its rough surfaces, this distinguishing feature demonstrates the material's capacity to provide strong particulate-matrix interface adhesion with the aluminium, which will aid in improving the wettability with the matrix and, as a result, the mechanical qualities of the resulting composite^39^. The EDS of the CCP is shown in Fig. 2f, and it displays silicon (Si), carbon (C), and oxygen (O) peaks (Si). These components, which are the main components of carbonated coal, SiO_2_ and graphite, proved their presence. The XRF and XRD analyses concur with this conclusion.

### X-ray fluorescence of the constituting materials

By using XRF analysis, the Al, PP, and CCP powders that make up the Al-PP-CCP hybrid composites were subjected to chemical analysis. The findings are shown in Tables 3 and 4.

**Table 3:** Aluminium's chemical composition.

Element	Al	Si	Fe	Cu	Mn	Mg	Zn	Cr	Ti	Ca	Others
wt%	97.743	0.61	0.44	0.16	0.024	0.821	0.0015	0.07	0.01	0.046	0.0745

**Table 4:** Chemical composition of PP and CCP.

Chemical compound	PP	CCP
SiO_2_	45.63	41.22
V_2_O_5_	0.15	0.24
Cr_2_O_3_	0.01	0.26
MnO	0.24	0.36
Fe_2_O_3_	17.24	12.75
Co_3_O_4_	0.09	0.09
Nb_2_O_3_	0.05	0.04
MgO	ND	4.78
CuO	0.06	0.21
P_2_O_5_	0.84	ND
SO_3_	0.14	13.32
CaO	9.83	3.55
K_2_O	5.37	1.21
BaO	0.26	0.22
Al_2_O_3_	15.53	13.42
Ta_2_O_5_	0.02	0.04
TiO_2_	3.54	5.93
Cl	0.73	1.85
ZrO_2_	0.21	0.35
Ag_2_O	0.003	0.04

The result shown in Table 3 revealed that the alloy has a significant amount of aluminium (98.18% of its weight), which is consistent with other research that has looked at alloy composites made of aluminium^40,41^. Additionally, the XRF study shown in Table 4 indicated that the primary elements of the CCP were SiO_2_, Al_2_O_3_, SO_3_, Fe_2_O_3_, TiO_2_, MgO, and CaO, whereas the major constituents of the PP were SiO_2_, Fe_2_O_3_, Al_2_O_3_, CaO, K_2_O, and TiO. The findings of^38,42^ for PP, and^43^ for CCP are consistent with this outcome. Additionally, the XRF study revealed that the chemical makeup of PP and CCP is comparable to other agricultural wastes utilized in metal matrix composites at the moment, such as bagasse, locust bean waste ash, rice husk ash, and fly ash^38,40,44^. Silica, iron oxide, and alumina are among the hardest materials, and^38^ claim that they are appropriate for reinforcing in a variety of metal matrixes due to their presence in PP and CCP.

### X-ray powder diffraction of the constituting materials

**Figure 3:**
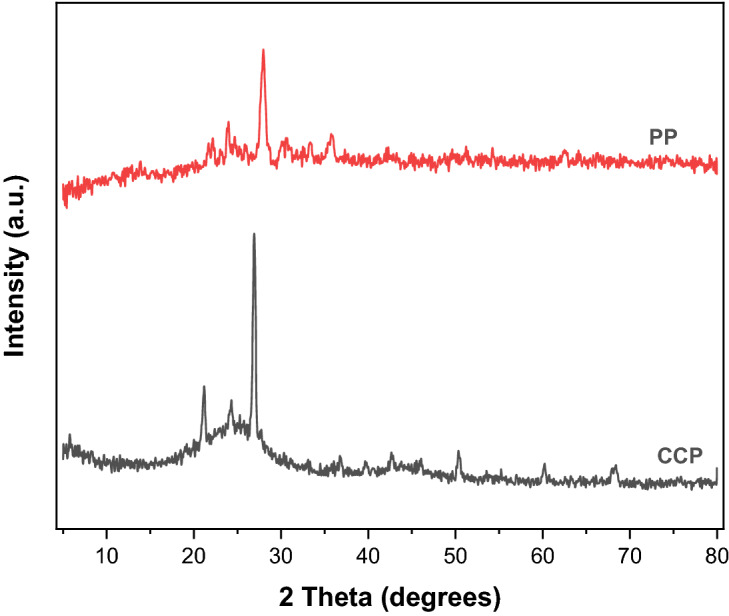
XRD analysis of PP and CCP powders.

Figure 3 displays the outcomes of the XRD characterisation of PP and CCP. The outcome displays the distinctive pumice peaks at 23° and 28° that belonged to the naturally occurring zeolite material dachiardite [(Ca, Na, K, Mg)_4_, (Si, Al)_24_O_48_,13H_2_O], anorthite (CaAl_2_Si_2_O_8_) albite (NaAlSi_3_O_8_), chlorite-serpentine. Additionally, the background line increased in the 20° to 30° 2Theta range, indicating the existence of amorphous quartz material in PP^45^. The CCP powder was matched, and the results revealed the existence of quartz, graphite, montmorillonite, muscovite, and chlorite phases.

### Percentage elongation and its optimization of stir casting process parameters

Based on the experimental runs associated with this study, as shown in Tables 1, 5 gives the percentage elongation of the Al-PP-CCP hybrid composites produced as well as the control Al-alloy’s percentage elongation. The result showed that the maximum percentage elongation of 5.6% was obtained at 2.5 wt% PP, 2.5 wt% CC, 200 rpm stirring speed, 700 °C processing temperature, and 5 min stirring time. A minimum percentage elongation of 2.09% was obtained at 10 wt% PP, 2.5 wt% CCP, 500 rpm stirring speed, 750 °C processing temperature, and 15 min stirring time. From this result, it can be inferred that the percentage elongation of the Al-PP-CCP hybrid composite decreases compared with the cast aluminium which has a percentage elongation of 7.51%. The decrease in the percentage elongation can be associated with the presence of hard and brittle reinforcement in the ductile Al matrix, resulting to a barrier in the plastic flow of the composite. Similar studies by^46–48^ among others, agreed with this finding.

**Table 5:** Experimental result for percentage elongation of the Al-PP-CCP hybrid composite and the S/N ratio.

Experimental run	Factors					Average percentage elongation (%)	S/N ratio
	PP (wt%)	CC (wt%)	SS (rpm)	PT (°C)	ST (min)		
1	2.5	2.5	200	700	5	5.60	14.96
2	2.5	5	300	750	10	4.68	13.40
3	2.5	7.5	400	800	15	4.57	13.20
4	2.5	10	500	850	20	3.09	9.80
5	5	2.5	300	800	20	3.30	10.37
6	5	5	200	850	15	3.81	11.62
7	5	7.5	500	700	10	4.82	13.66
8	5	10	400	750	5	3.80	11.60
9	7.5	2.5	400	850	10	2.50	7.96
10	7.5	5	500	800	5	2.48	7.89
11	7.5	7.5	200	750	20	4.20	12.47
12	7.5	10	300	700	15	4.17	12.40
13	10	2.5	500	750	15	2.09	6.40
14	10	5	400	700	20	2.72	8.69
15	10	7.5	300	850	5	2.38	7.53
16	10	10	200	800	10	3.49	10.86
PE Average						**3.61**	
Control						7.51	

#### Optimization of stir casting process parameters

Using the Taguchi design technique, the stir casting process parameters were adjusted based on the S/N ratio of the experimental runs shown in Table 5. The response table for the S/N ratio and the mean, depending on the various factors and levels taken into consideration in this investigation, is shown in Table 6. The percentage elongation of the Al-PP-CCP hybrid composites is shown to be influenced by each stir casting process parameter in this result, which also ranks the factors based on the S/N ratio to illustrate which factor has the most impact. As a consequence, the PP has the greatest effects, followed by processing temperature, stirring speed, CCP, and stirring time.

**Table 6:** Response table for the percentage elongation.

Level	Pumice particles (wt%)		Carbonated coal particles (wt%)		Stirring speed (rpm)		Processing temperature (°C)		Stirring time (min)	
	Mean (%)	S/N (dB)	Mean (%)	S/N (dB)	Mean ((%)	S/N (dB)	Mean (%)	S/N (dB)	Mean (%)	S/N (dB)
1	4.49	12.84	3.37	9.92	4.28	12.48	4.33	12.43	3.57	10.50
2	3.93	11.81	3.42	10.40	3.63	10.93	3.69	10.97	3.87	11.47
3	3.34	10.18	3.99	11.72	3.40	10.36	3.46	10.58	3.66	10.91
4	2.67	8.37	3.64	11.16	3.12	9.44	2.95	9.23	3.33	10.33
Delta	1.82	4.47	0.62	1.79	1.16	3.04	1.38	3.20	0.55	1.14
Rank	1	1	4	4	3	3	2	2	5	5

#### Effect of stir casting process parameters on the percentage elongation

**Figure 4:**
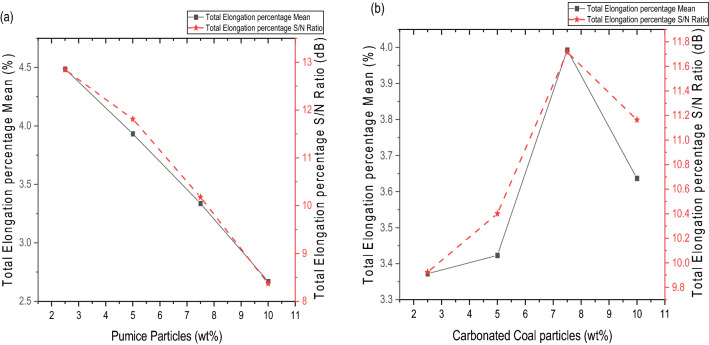
Variation of percentage elongation with reinforcement addition; (**a**) pumice powder vs percentage elongation, (**b**) carbonated coal particles vs percentage elongation.

The effect of PP, CCP, stirring speed, processing temperature, and stirring time on the percentage elongation is shown in Fig. 4. Figure 4a shows the effect of PP on the percentage elongation of the reinforced aluminium metal matrix composite. It was observed that the percentage elongation decreased as the weight composition of PP increased from the maximum of 2.5 to 10 wt%. The decrease in percentage elongation can be attributed to the presence of harder and stiffer pumice reinforcement, which contains quartz, anorthite, and albite, as shown in the XRF analysis. This finding is consistent with the studies by Nagaral et al.^49^ and Anbuchezhiyan et al.^50^, who observed that the percentage elongation of metal matrix composites decreases by increasing the reinforcement content.

Figure 4b shows CCP’s effect on the reinforced Al-PP-CCP hybrid composite's percentage elongation, and it was observed that the percentage elongation of the composite increases 3.38% to 3.99% with an increase in the weight composition of CCP from 2.5 wt% to 7.5 wt%, and beyond this point, the trend reverses. The increase in strength can be attributed to the homogenous dispersion of the CCP reinforcement in the matrix. The reverse in the percentage elongation obtained after 7.5 wt% is attributed to the poor wettability that increases with an increase in the weight composition of CCP. This finding is similar to the work of Muni et al.^51^, which observed an increase in percentage elongation as a result of the uniform dispersion of the reinforcement.

**Figure 5:**
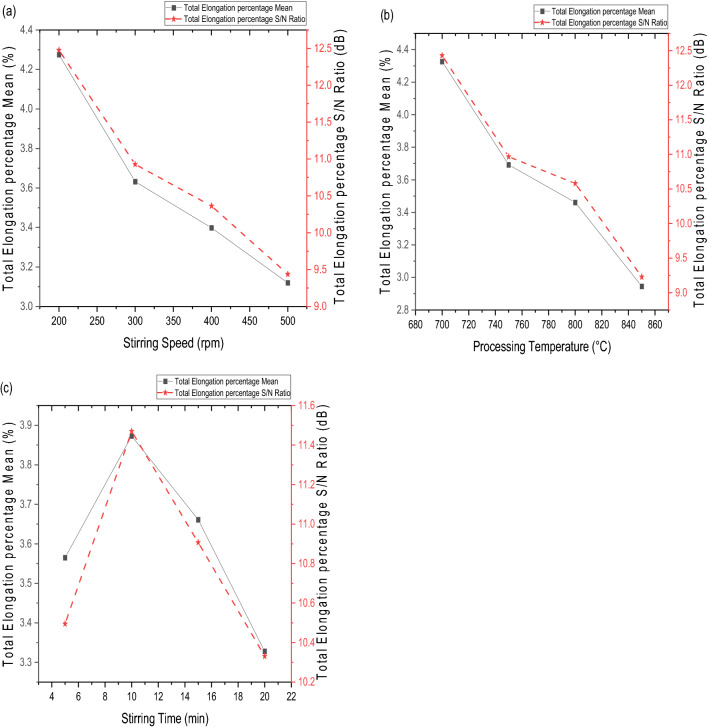
Variation of percentage elongation with stirring process parameter; (**a**) stirring speed vs percentage elongation, (**b**) processing temperature vs percentage elongation (**c**) stirring time vs percentage elongation.

Figure 5a shows the effect of stirring speed on the percentage elongation of the reinforced aluminium metal matrix composite. It was observed that as the stirring speed increases, the percentage elongation decreases from a maximum value of 4.275% at a stirring speed of 200 rpm to a minimum of 3.12% at a stirring speed of 500 rpm. The decrease can be attributed to increased agitation severity of the slurry, resulting in clustering of the reinforcement particles and promoting the entrapment of gases into the slurry, which causes high porosity and blow holes. These results agree with some related studies by^52^, who observed that higher stirring speeds imposed a considerable non-uniformity in the particle distribution due to the increased agitation severity of the slurry, resulting in clustering of the particles and gas absorption into the slurry.

Figure 5b shows that by increasing the pouring temperature, percentage elongation decreased from 4.327% at 700 °C to 2.945% at 850 °C. The decrease can be attributed to the non-uniform distribution of the reinforcement in the slurry and the absorption of gases into the melt, which can cause porosity. Stirring at a high temperature above the optimal value might also be the cause of reduction because stirring at a higher temperature will result in the formation of detrimental new intermetallic phases and blow holes in the fabricated composite. This finding is consistent with the studies conducted by^53^, who observed that an increase in the stirring temperature above the optimum led to a less homogeneous distribution of the particles and the formation of undesirable phases, which have an adverse effect on the composite.

The variation of percentage elongation with stirring time (ST) for the Al-PP-CCP hybrid composite is shown in Fig. 5c. From the result, it was observed that the percentage elongation increased to an optimal value of 3.87% at a stirring time of 15 min and then kept decreasing till it got to the stirring time of 20 min. The increase can be attributed to the excellent mixing of the reinforcements, which led to its fair distribution in the matrix. In contrast, the decrease in the percentage elongation with an increase in the stirring time can be attributed to a longer stirring duration, which leads to gas absorption and oxidation in the liquid aluminium matrix. This result is consistent with the studies by Azadi et al.^54^ who observed that increasing stirring time duration above the optimum value will undoubtedly increase gas absorbability and oxidation of the prepared composites, which will decrease their mechanical properties.

#### Interactions contour plots

**Figure 6:**
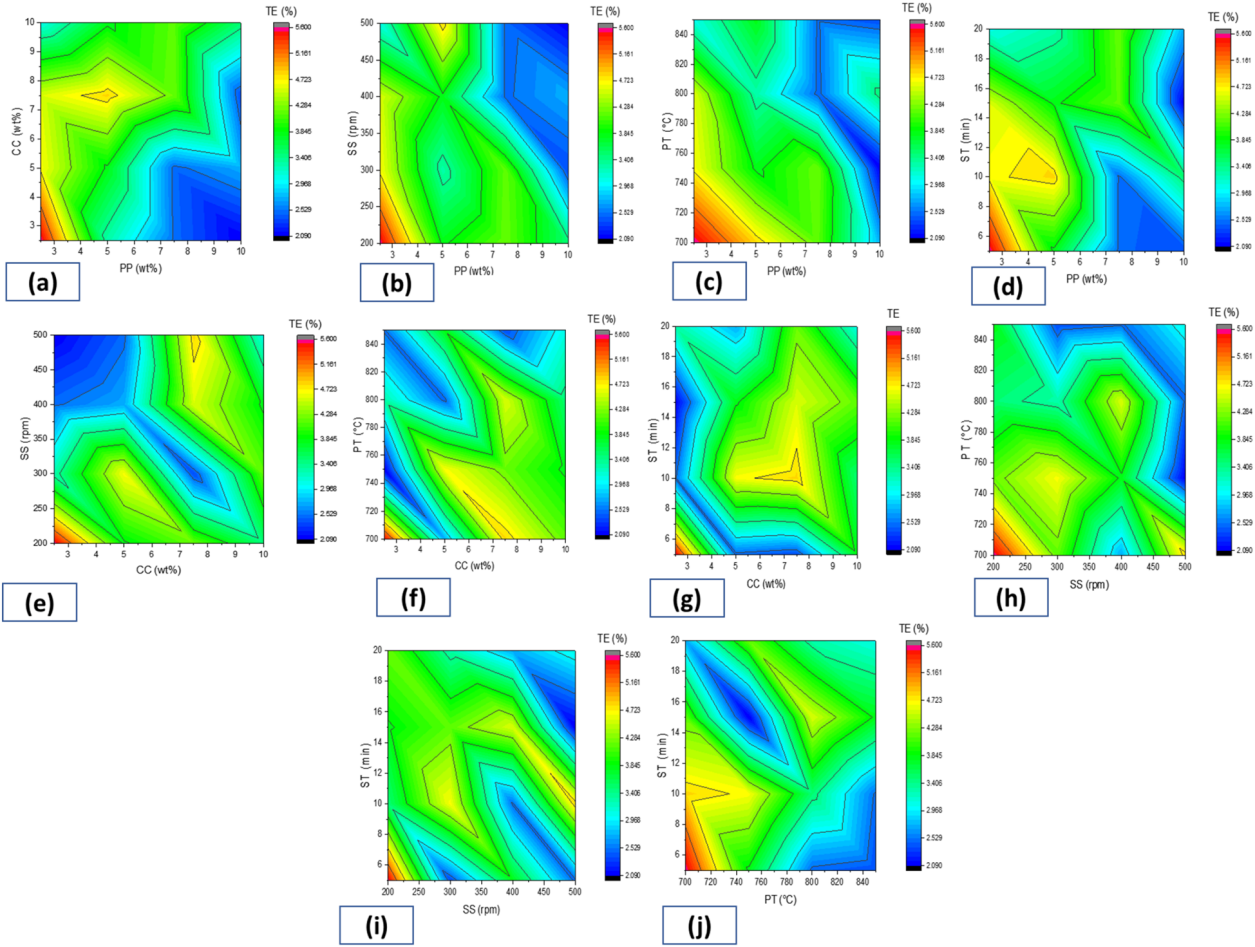
Interaction Contour Plots of Percentage Elongation (PE) with Process Parameters at Various Reinforcement Addition: (**a**) PE vs.CC, PP, (**b**) PE vs. PT, PP, (**c**) PE vs. SS, PP, (**d**) PE vs. ST, PP, (**e**) PE vs. SS, CC, (**f**) PE vs.PT, CC, (**g**) PE vs.ST, CC, (**h**) PE vs. PT, SS, (**i**) PE vs. ST, SS, (**j**) PE vs. PT, ST.

To examine the relationship between the response variable and two control variables, contour plots were used as given in Fig. 6.

It was observed in Fig. 6a that, while keeping other process parameters constant, a maximum percentage elongation of 5.6% can be obtained through an interaction between PP at 2.5 wt% and CCP at 2.5 wt%. Figure 6b shows that a maximum percentage elongation of 5.6% can be obtained through an interaction of PP at 2.5 wt% and PT at 700 °C. Figure 6c shows that the interaction of 2.5 wt% PP and a SS of 200 rpm gives a maximum percentage elongation of 5.6%. As shown in Fig. 6d, the maximum percentage elongation of 5.6% can be obtained at the interaction of 2.5 wt% of PP content and 5 min ST duration. Figure 6e indicated that at the interaction of 2.5 wt% of CC content and 200 rpm of SS, maximum percentage elongation of 5.6% can be obtained. Figure 6f observed that a maximum percentage elongation of 5.6% can be obtained at an interaction of 2.5 wt% of CCP and 700 °C processing temperature. Figure 6g shows that the maximum percentage elongation of 5.6% can be obtained with an interaction of 2.5 wt% CCP and an ST of 5 min. Figure 6h shows that a higher percentage elongation of 5.6% could be attained at a stirring speed of 200 rpm and a processing temperature of 700 °C. Figure 6(i) shows that to obtain maximum percentage elongation of 5.6%, the slurry must be stirred at 200 rpm for 5 min, keeping other parameters constant. Figure 6j shows that a maximum percentage elongation of 5.6% could be obtained at the interaction of processing temperature at 700 °C and string time of 5 min if other parameters are kept constant.

#### Optimum combination for percentage elongation

From Table 6 and Figs. 4 and 5, the highest mean S/N ratios obtained for percentage elongation in terms of stir casting process parameters are PP at 2.5 wt%, CCP at 7.5 wt%, stirring speed at 200 rpm, processing temperature at 700 °C, and stirring time at 10 min, corresponding to PP_1_–CC_3_–SS_1_–PT_1_–ST_2_. Using the optimal settings of the stir casting process parameter (PP_1_–CC_3_–SS_1_–PT_1_–ST_2_), an optimal percentage elongation for the hybrid aluminium composite can be predicted using Eq. (2) and Table 6.

2 $${T}_{opt}={T}_{m}+{\sum }_{i=1}^{{k}_{n}}(({T}_{ik}{)}_{max}-{T}_{m})$$

where T_m_ is the overall mean or S/N ratio obtained from Table 5, T_m_ = 3.6065%; ${(T}_{ik}{)}_{max}$ is the mean at optimum level i of factor k , T_1PP_ = 4.486%, T_3CC_ = 3.993%, T_1SS_ = 4.275%, T_1PT_ = 4.327%, and T_2ST_ = 3.873% bolded in Table 6, and ${k}_{n}$ is the number of main design factors that affect the response, which is equal to 5. This produced the optimal percentage elongation of 6.51%.

To validate the Taguchi predicted optimum conditions obtained; a new Al-PP-CCP composite was cast using the optimum levels of the factors (PP_1_–CC_3_–SS_1_–PT_1_–ST_2_), and percentage elongation confirmation tests were conducted as per ASTM standard on the produced sample with three replications; the results are shown in Table 7. The confirmation percentage elongation resulted in a value of 7.12%.

**Table 7:** Confirmatory results comparison at the optimal level.

	Optimal process parameter settings	Predicting value (%)	Experimental value (%)	% Error
S/N ratio (dB)	PP_1_–CC_3_–SS_1_–PT_1_–ST_2_	16.28	17.05	4.53
PE (%)		6.51	7.12	8.51

#### Regression analysis of percentage elongation

Linear regression analysis was performed using Minitab software. This analysis generated an ANOVA that considered the factors and their interactions to determine the significance level of each processing parameter. The obtained result is shown in Table 8 in which at the significant level of 0.05, the regression model, PP, SS, and PT are significant, and PP has the highest percentage contribution to the percentage elongation of the hybrid composite. The regression analysis produced a predictive mathematical model for the percentage elongation (PE) as a function of the stir casting process parameters that gave a high level of prediction, with R-Square, R-Square (adj) and R-Square (pred) values of 91.60%, 87.41%, and 79.32%, respectively. According to^55,56^, an R-Square value greater than 75% is deemed adequate, implying a good fit between the responses and the process parameters. The regression model is given in Eq. (3).

**Table 8:** Regression analysis of variance for the percentage elongation of hybrid composite.

Source	DF	Adj SS	Adj MS	F-Value	P-Value	
Regression	5	14.4185	2.884	21.82	0.00	
PP	1	7.3024	7.302	55.26	0.00	46.39
CC	1	0.3731	0.373	2.82	0.12	2.37
SS	1	2.7368	2.737	20.71	0.00	17.39
PT	1	3.8354	3.835	29.02	0.00	24.37
ST	1	0.1708	0.171	1.29	0.28	1.09
Error	10	1.3215	0.132			8.40
Total	15	15.74				

3 $$PE=13.09-0.2417 PP+0.0546 CC-0.003699 SS-0.00876 PT-0.0185 ST$$

**Figure 7:**
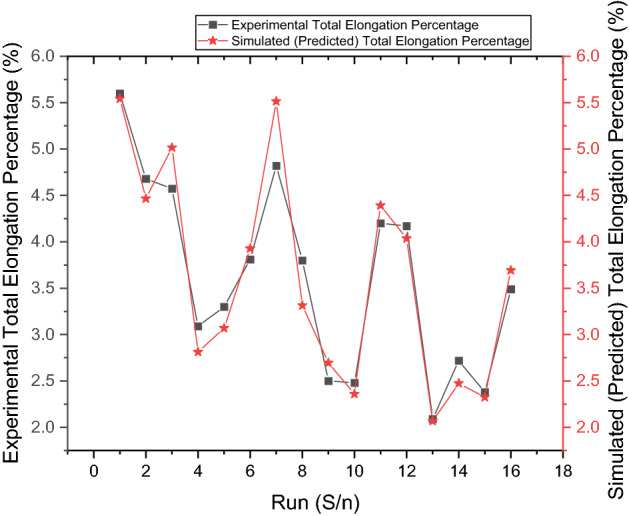
Predicted vs. Experimental plot of percentage elongation of the Al-PP-CCP hybrid composite.

Figure 7 compares the predicted and experimental percentage elongation of the experimental runs considered in this study. From the comparison of these graphs within the confidence interval (CI) range of the model’s prediction, it shows the acceptability of the optimum percentage elongation prediction within the confidence interval of 95%.

##### Confidence interval (CI)

For this study, the experimental value is expected to fall within this range;

$${\text{PE}}_{{{\text{predictive}}}} - {\text{CI}} < {\text{PE}}_{{{\text{experimental}}}} < {\text{PE}}_{{{\text{predictive}}}} + {\text{CI}}$$

where TS_predictive_ is the predicted or optimum total elongation percentage, TS_experimental_ is the experimental value after the confirmation test, and while CI is the Confidence Interval.

Equation (4) was used to evaluate the confidence interval.

4 $$C.I.=\sqrt{{f}_{\propto (1,{d}_{e})}{v}_{e}\left(\frac{1}{U}+\frac{1}{W}\right)}$$

where ${F}_{\alpha }\left(1, {F}_{e}\right)$= F ratio required for α = risk ; F_e_ = error DOF; from table 4.5 F_e_ = 10 ∴ from F-table ${F}_{\alpha }\left(1, {F}_{e}\right)$= ${F}_{0.05}$(1,10) = 4.96, V_e_ = error variance, from the ANOVA Table 8 V_e_ = 1.3215; W = same as the number of replications to run confirmation test = 3; U = effective number of replications.

5 $$U=\frac{N}{1+T}$$

where N is the total number of results = 48 and T is total degree of freedom of the controlled factors = 5. Substituting these values into Eq. (5).

$$\therefore U=$$ 8.

Substituting the values of ${f}_{\alpha \left(1, de\right)}, {v}_{e}, U, and w$, into Eq. (4)

∴ C.I = 1.733.

The obtained total elongation percentage value from the confirmatory test shows that the experimental value lies within the confidence interval range of the total elongation percentage, such that:

$${\text{PE}}_{{{\text{predictive}}}} - {\text{CI}} < {7}.{12} < {\text{PE}}_{{{\text{predictive}}}} + {\text{CI}}$$

$${4}.{7949} < {\text{PE}}_{{{\text{experimental}}}} < {8}.{2614}$$

In this case, PE_predictive_ = 6.51.

## Conclusion

The study took into account the use of the Taguchi optimization approach to optimize the weight composition of natural ceramic reinforcement (pumice and carbonated coal particles) as well as the impact of stir casting process parameters on the percentage elongation of Al-PP-CCP hybrid composites. The hardest constituents, silica, iron oxide, and alumina, were found during the reinforcement's characterisation, indicating that PP and CCP are suitable for use as reinforcement in a variety of metal matrixes, though they decrease the percentage elongation property. Utilizing stir casting process parameter optimization, it was discovered that the PP has the greatest impact on the hybrid composite's percentage elongation, followed by processing temperature, stirring speed, CCP, and stirring duration. Due to the presence of tougher and stiffer PP reinforcement, it was noticed that the percentage elongation rose as the weight composition of PP dropped, reaching a maximum at 2.5 wt%. The best percentage of elongation was found to be 5.6%, which is 25.43% less than the percentage elongation of Al-alloy without reinforcement, at 2.5 wt% PP, 2.5 wt% CCP, 700 °C PT, 200 rpm SS, and 5 min ST time. The obtained percentage elongation value from the confirmatory test shows that the experimental value lies between the confidence interval range of the percentage elongation. With R-Square and R-Square (adj) values of 91.60% and 87.41%, respectively, the regression analysis established a predictive mathematical model for the percentage elongation (PE) as a function of the stir casting process parameters and provided a high degree of prediction.

## Scope of feature work

Evaluating the Tribological and thermal properties of the hybrid composite.

## Data Availability

The raw/processed data required to reproduce these findings cannot be shared at this time as the data also forms part of an ongoing study (PhD Thesis).
